# Potential Effect of Dietary Supplementation of Tannin-Rich Forage on Mitigation of Greenhouse Gas Production, Defaunation and Rumen Function

**DOI:** 10.3390/vetsci10070467

**Published:** 2023-07-17

**Authors:** Néstor Acosta-Lozano, Marcos Barros-Rodríguez, Carlos Guishca-Cunuhay, Veronica Andrade-Yucailla, Katherine Contreras-Barros, Carlos Sandoval-Castro, Mona Mohamad Mohamad Yasseen Elghandour, Abdelfattah Zeidan Mohamed Salem

**Affiliations:** 1 Centro de Investigaciones Agropecuarias, Facultad de Ciencias Agrarias, Universidad Estatal Península de Santa Elena, La Libertad 240204, Ecuador; nacosta@upse.edu.ec (N.A.-L.); crisita_2725@hotmail.com (V.A.-Y.); 2 Facultad de Ciencias Agropecuarias, Universidad Técnica de Ambato, Sector el Tambo-La Universidad, vía a Quero, Cevallos 1801334, Ecuador; 3 Department of Animal Nutrition and Rumen Biotechnology, Ruminant Feedlot Ranch-PROCESA, Street Playita-Estero Hondo, La Mana 050202, Ecuador; guishcacarlos@gmail.com; 4 Facultad de Ciencias Biológicas, Universidad Central del Ecuador, Campus El Dorado-Itchimbía, Quito 170403, Ecuador; katherine_ecb20@hotmail.com; 5 Departamento de Nutrición Animal, Facultad de Medicina Veterinaria y Zootecnia, Universidad Autónoma de Yucatán, Carretera Mérida-Xmatkuil Km. 15.5. Apdo. 4-116 Itzimná, Mérida 97100, Mexico; carlos.sandoval@correo.uady.mx; 6 Facultad de Medicina Veterinaria y Zootecnia, Universidad Autónoma del Estado de México, Toluca 50200, Mexico; mmohamede@uaemex.mx (M.M.M.Y.E.); salem@uaemex.mx (A.Z.M.S.)

**Keywords:** *Acacia mearnsii* leaves, tannins, rumen degradation, protozoa, gas production, methane

## Abstract

**Simple Summary:**

In tropical and subtropical regions, low availability and poor nutritional quality of forage sources, limit ruminant productivity and promote enteric production of CH_4_ and CO_2_ which are considered greenhouse gases (GHG). Non-conventional trees and shrubs, as forage resources, are considered an effective alternative for the improvement of animal performance as they possess good nutritional value along with their content of secondary metabolites. The aim of this experiment was to assess the effect of including *Acacia mearnsii* leaves in highly fibrous rations (corn stover) and its effect on rumen degradation kinetics, digestibility, microbial synthesis and in vitro production of gas, CH_4_ and CO_2_. It is concluded that up to 15% of *A. mearnsii* leaves can be recommended in the diet of ruminants as it causes a reduction in the population of protozoa (holotrich and entodiniomorph), as well as the production of CH_4_, without generating adverse effects on the ruminal degradation kinetics, nutrient digestibility and microbial protein production.

**Abstract:**

This experiment evaluated the effect of including *Acacia mearnsii* leaves in a high-fiber diet (corn stover), on ruminal degradation kinetics, digestibility, microbial biomass production, and gas, CH_4_, and CO_2_ production. Four experimental diets were tested, including a control with 100% corn stover (T1), and three additional diets with corn stover supplemented at 15% *A. mearnsii* leaves (T2), 30% *A. mearnsii* leaves (T3) and 45% of *A. mearnsii* leaves (T4). The highest dry matter in situ degradation (*p* ≤ 0.001) and in vitro digestibility (*p* ≤ 0.001) was found in T1 (80.6 and 53.4%, respectively) and T2 (76.4 and 49.6%, respectively) diets. A higher population of holotrich and entodiniomorph ruminal protozoa was found (*p* = 0.0001) in T1 at 12 and 24 h. Diets of T1 and T2 promoted a higher (*p* = 0.0001) microbial protein production (314.5 and 321.1 mg/0.5 g DM, respectively). Furthermore, a lower amount of CH_4_ was found (*p* < 0.05) with T2, T3 and T4. It is concluded that it is possible to supplement up to 15% of *A. mearnsii* leaves (30.5 g TC/kg DM) in ruminant’s diets. This decreased the population of protozoa (holotrich and entodiniomorph) as well as the CH_4_ production by 35.8 and 18.5%, respectively, without generating adverse effects on the ruminal degradation kinetics, nutrient digestibility and microbial protein production.

## 1. Introduction

Ruminant production in tropical and subtropical areas is generally based on pasture grazing with a predominance of grasses [1,2,3,4], characterized by low amounts of protein (<7%), a high proportion of structural carbohydrates (60–80%) [5,6] and low digestibility (<50%) [7]. Characteristics that affect the productivity of animals, in response to the consumption of poor quality forage and the subsequent production of greenhouse gases (GHG) (mainly methane (CH_4_), carbon dioxide (CO_2_) and nitrous oxide (N_2_O)), as a result of the metabolism of structural carbohydrates, gases that are later eliminated through burping or flatulence and generate considerable energy losses in the animal (2 to 12%) [8,9,10,11]. Ruminants are responsible for producing approximately 47% of CH_4_, 29% of N_2_O, and 27% of CO_2_ of total GHG (14.5%) of anthropogenic origin from livestock activity, so the search for possible solutions is very important [12,13,14,15]. As an alternative to solve these problems, the use or incorporation of tree fodder with anti-methanogenic capacity in ruminant feeding is proposed [16]. This can be integrated into production systems such as protein banks (cut and carry) or under silvopastoral systems [17,18], due to; (i) potential nutritional value (protein: 15.8% to 33.3%, NDF: 26.2% to 33.3%, FDA: 14.8% to 22.8%) [19], (ii) high digestibility (53.1% to 82.5%) [20], and (iii) its content of secondary metabolites. These compounds are capable of modulating fermentation in the rumen and reducing CH_4_ production, a desired effect due to its potential for environmental damage (26–28 times greater than CO_2_) [21,22].

The CH_4_ reduction when trees or shrubs are incorporated into ruminant feed has been attributed to a condensed tannins (CT) effect on the community of methanogenic archaea and protozoa at the ruminal level [23], probably due to their reduced growth or activity due to inhibition of the exchange of dihydrogen (H_2_) between species (methanogen-protozoan) as a result of changes in cell membrane permeability, reduction in the availability of nutrients (mainly protein) by forming tannin–protein complexes and inhibiting the development of ruminal microorganisms and interruption in the process of reducing CO_2_ to CH_4_ due to the decrease in H_2_ available in the rumen [24]. On the other hand, tannins can also improve the use of protein, effects attributed mainly to CT that, forms complexes with proteins (tannin–protein complex) which increases the amount of by-pass protein to the duodenum, later evidencing better productive yields in animals [25]. However, the greatest limitation in the use of CT is reduced microbial growth and hence a reduced protein supply to the animal [26]. However, it has been reported that the effect of tannin will depend on CT type, origin, dose, molecular weight and adaptation of the animals to its intake [27,28]. Detrimental effects on animal health show in performance, intake and digestibility. This is probably due to: (a) consumption of tannins in amounts higher than 55 g CT/kg DM [29]; (b) decreased enzyme activity such as trypsin and amylase [30]; (c) reduction in food consumption due to decreased palatability as a result of the effect of CT on salivary glycoproteins [31]; and (d) decreased digestion of nutrients (carbohydrates, protein and lipids) [32].

Thus, using *Acacia mearnsii* as forage for ruminants is a valuable alternative due to its wide distribution around the world and its ability to adapt to temperatures between 14.7–27.8 °C, as well as for presenting considerable quantities of bioactive compounds, mainly CT (35% to 45%), due to the antimethanogenic effect that this compound can provide [33]. Previous research has shown variability in the tannin effects on ruminal fermentation. Waghorn et al. [32] showed in their study a higher weight gain and milk production (30% and 11%, respectively) in small ruminants fed tannin rich forages. However, Vargas-Ortiz et al. [33] found negative effects on the degradation and digestibility of DM and OM when incorporating *A. mearnsii* forage in non-fibrous diets containing *Lolium perenne* and *Medicago sativa*. Similarly, Ávila et al. [34] mention the absence of effects on the protozoa population when using extract of *A. mearnsii* rich in CT in diets high in grain (Tifton 85 hay (60%), ground corn (30.7% to 33.7%) and soybean meal (52.1% to 58.4%)). Therefore, the aim of this research was to evaluate the effect of the incorporation of *A. Mearnsii* leaves on rumen degradation kinetics, digestibility, microbial synthesis and in vitro fermentation (gas, CH_4_ and CO_2_ production).

## 2. Materials and Methods

### 2.1. Study Location

The present research was carried out at “Querochaca” Experimental Farm and Rumenology Laboratory of the Universidad Técnica de Ambato, Facultad de Ciencias Agropecuarias, Tungurahua, Ecuador, at an altitude of 2890 m above sea level. In the sector, there are maximum temperatures of 20 °C and minimum of 7 °C and an average ambient temperature of 15 °C.

### 2.2. Forage Samples and Treatments

The corn stover (*Zea mays*) (2-hectare crop) was cut 5 cm from the ground after harvesting the cob at the age of 5 months, approximately 50 kg of plants were crushed in a forage grinder (CREMASCO, model DP2, Brasilia, Brazil). A subsample (5 kg) of the minced material was dried in a forced air oven at 55 °C for 72 h to estimate dry matter (DM). The dry corn forage was ground in a hammer mill with a 2 mm sieve size, once ground it was passed through a 1 mm sieve to standardize its particle size and perform both in situ and in vitro tests.

*Acacia mearnsii* leaves were collected from 15 trees that are planted in the Faculty of Agricultural Sciences—UTA, Ecuador. The forage collection was carried out taking young and mature leaves. Subsequently, the forage (50 kg) was dried in a greenhouse and a 1 kg subsample was oven dried at 60 °C to determine DM and later used for chemical composition analysis. The dehydrated forage was ground under the methodology described for corn stover (see above).

The dry and sifted forages were mixed to form the following treatments (expressed in % DM); T1: 100% corn stover, T2: 85% corn stover + 15% *A. mearnsii* leaves, T3: 70% corn stover + 30% *A. mearnsii* leaves, T4: 65% corn stover + 45% *A. mearnsii* leaves. Chemical composition of experimental treatments is presented in Table 1.

### 2.3. In Situ Rumen Degradation, In Vitro Digestibility and Microbial Biomass Production

The DM degradability was measured by means of the nylon bag technique (0.42 µ) [35]. Five castrated male bovines (450 ± 30 kg LW) were used. Animals were fitted with ruminal cannula (Bar Diamond, Parma, ID, USA). The animals housed individually and fed ad libitum a ration containing alfalfa forage (*Medicago sativa*). Water was also provided *ad libitum*. Four grams of DM were incubated in each bag (times: 0, 6, 12, 24, 36, 48, 72 and 96 h). After incubation, bags were washed with tap water and placed in an oven at 60 °C. Degradation was calculated as a ratio of incubated and residual material. The data was fitted to the exponential equation: Y = a + b (1 − e^−ct^) and the effective degradation was calculated as ED = a + [(b × c)/(c + k)] at 2, 5 and 8% passage rate [36].

Dry matter digestibility was performed in vitro, the liquid and solid fractions of rumen content were obtained separately from each of the five individual bulls (each one represents a replicate). Ruminal content was obtained before morning feeding, kept in a sealed plastic container (at 39 °C) and immediately transported to the laboratory (within 1 h of collection). A N-rich medium was prepared [37]. For each treatment, five amber glass bottles (replicates) of 100 mL capacity were used with 60 mL of the inoculum (70:30 medium; artificial saliva/inoculum; rumen content), containing 0.5 g of sample for each treatment at each time and five additional bottles without feed sample were used as blanks. The bottles were incubated between 39–40 °C. At the end of the incubation period (48 h), the in vitro DM digestibility was calculated from the difference between the incubated DM and the residue obtained afterwards by filtering the residues. DM digestibility was corrected with the blank residual.

The microbial biomass production (MBP) was calculated using the equation proposed by Blümmel et al. [38], where: MBP (mg) = truly degraded substrate of the OM − (gas volume (mL) × stoichiometric factor (2.2; for forage fermentation)).

### 2.4. Protozoa Population

Five glass bottles were prepared by the same procedure used for in vitro digestibility (see above). A one mL post-incubation sample was taken at 12 and 24 h and stored in an eppendorf tube with a 2 mL capacity. The ruminal content samples were preserved with a drop of formalin and kept at 4 °C until the protozoa were quantified using an optical microscope with 40× objective lenses and a Fuchs–Rosenthal camera (depth: 0.2 mm, small square area: 0.0625 mm^2^). The number of ciliate protozoa was reported as Log_10_ of total number + 1 per mL of rumen liquor. To determine the population of protozoa, they were stained with a solution of methyl formamide green according to the methodology described by Ogimoto and Imai [39].

### 2.5. Gas, CH_4_ and CO_2_ Production

Gas production was measured according to the methodology described by Theodorou et al. [40]. Ruminal fluid and nitrogen-rich medium were prepared as mentioned before for in vitro digestibility. For each treatment, 0.5 g of DM was placed in a glass bottle (100 mL nominal capacity). Then, 60 mL of ruminal inoculum (70:30 artificial saliva/ruminal inoculum) were added under a constant CO_2_ flow. Bottles were sealed and incubated at 39–40 °C. Gas pressure and volume were measured manually at 3, 6, 9, 12, 24, 36, and 48 h with a DELTA OHM model DO 9704 pressure transducer (Delta, model OHM Srl, Padova, Italy) and plastic syringes. Methane and CO_2_ production was measured with the help of a SMART 6 SENSOR SAMPLE DRAW GAS MONITOR GX-6000 gas meter, UK according to the methodology described by Elghandour et al. [21]. Total gas, CH_4_ and CO_2_ production was estimated from 0.5 g fermented DM. The gas data (mLgas/g fermented DM) were fitted to the equation y = D (1 − e^−kt^) described by Krishnamoorthy et al. [41], where: y = cumulative gas production at a given time (mL), D = potential cumulative gas production (mL/0.5 g fermented DM), k = rate of gas production (h–I) and t = time of fermentation (h). Parameters D and k were estimated by an iterative method of least squares using a nonlinear regression procedure of the Graphpad Prism 8 program (San Diego, CA, USA).

### 2.6. Chemical Analysis

The dry matter (DM) (# 7,007), ash (# 7,009) [42], Neutral detergent fiber (NDF), acid detergent fiber (ADF) (ANKOM Technology, Macedon, NY, USA. Methods 13 and 12, respectively), NDF was assayed with heat-stable alpha-amylase and sodium sulfite (Na_2_SO_3_) and expressed with residual ash (the latter also for ADF), CP (N × 6.25) (LECO CHN 628, LECO Corporation) and CT (catechin equivalent) [43] were determined.

### 2.7. Experimental Design and Statistical Analyzes

All variables were analyzed with ANOVA. A completely randomized design, with four treatments and five replicates was employed [44]. All means were contrasted with the Tukey test (SAS version 9.2, SAS Institute, Cary, NC, USA).

## 3. Results

### 3.1. Rumen Degradation, Digestibility and Microbial Biomass Production

Rumen degradation kinetics of DM showed differences (*p* < 0.05) between treatments (Table 2), with T3 and T4 having the lowest value in the insoluble but potentially degradable fraction (B), potential degradation (A + B) and effective degradation, in the different passage rates at 0.05 (45.70 and 42.70%, respectively) and 0.08 (41.00 and 38.80%, respectively), compared to the other treatments (T1 and T2).

For the DM digestibility and microbial biomass production, the diets of T1 and T2 had the highest value for digestibility versus T3 and T4 diets. The microbial biomass production was higher in the T1 and T2 diets, and lower in the T3 and T4 diets, with an average difference of 72.45 mg/0.5 g DM (Table 2).

### 3.2. Rumen Protozoa Population

Differences amongst the protozoan populations were found for all treatments (*p* < 0.05). The population of Holotrich and Entodiniomorph protozoa were lower (*p* = 0.0001) in the T4 treatment at 12 and 24 h post-incubation (0.5 and 0.0–1.6 and 1.1 protozoa: Log_10_, respectively) (Table 2).

### 3.3. Gas, CH_4_ and CO_2_ Production

Total gas production was lower (*p* = 0.0001) in diet T4, with a difference of 50.6 mL gas/0.5 g fermented DM compared to the treatment with higher gas production (diet T1). Regarding the gas production at different post-incubation hours, it was lower (*p* = 0.0002) at 48 h in T4 versus other diets. The CH_4_ production at 12 and 24 h was lower (*p* < 0.05) in diets T2, T3 and T4 and at 48-hours post-incubation, and it was lower (*p* = 0.0001) in diet T4 versus other treatments. With respect to CO_2_ production, differences were only observed at 24 h with T4 presenting the lowest values (*p* = 0.0001) (Table 3).

## 4. Discussion

### 4.1. Rumen Degradation, Digestibility and Microbial Biomass Production

CT can generate positive or negative effects on digestion and health in ruminants. This depends on the type, source, dose, molecular weight, and chemical composition of the diet and the adaptability of the animals to its consumption [27,28,45]. From these perspectives, the lower potential in situ degradation and in vitro digestibility of DM observed in the T3 and T4 diets (Table 2), is probably due to the increase in tannin levels in response to the increasing incorporation of *A. mearnsii* and its possible effect on bacteria that degrade nutrients (carbohydrates, proteins and lipids) [46]. In this study we observed the microbial biomass production decrease as the level of tannins exceeded the beneficial threshold in the diet, that is, beyond 55 g of CT/kg DM. This effect is probably attributed to (a) the antimicrobial activity of tannins, in response to structural changes induced in the cell wall of bacteria, attributed to the interaction of tannin with secreted extracellular enzymes, (b) alteration of the cell membrane, (c) inhibition of microbial metabolism, (d) nutrient deficit for the growth of bacteria and e) reduction in the availability of cations, essential for the subsistence of microorganisms [26].

However, the in vitro inhibition of the activity of the extracellular endoglucanase enzyme in *Fibrobacter succinogenes* has been described when using CT of *Lotus corniculatus* in doses higher than 100 μg/mL [47]. The CT of *Lotus corniculatus* and *Onobrychis viciifolia* have also shown inhibitory effects on fibrolytic and proteolytic microorganisms such as *F. succinogenes*, *Butyrivibrio fibrisolvens*, *Clostridium proteoclasticum*, *Eubacterium* spp., *Ruminococcus albus*, *Ruminobacter amylophilus* and *Streptococcus bovis* with the consequent reduction in proteolytic and fibrolytic activity [48,49]. Moreover, the low digestibility observed can also be attributed to the limited access of the microorganisms to the fiber, as a result of the possible formation of tannin–cellulose complexes and subsequent reduction in the enzymatic activity of the fibrolytic microorganisms [50]. This is evidenced in the decrease in ruminal degradation and digestion in this study in diets T1 and T2. Vargas-Ortiz et al. [33] and Kozloski et al. [51], also found low digestibility when incorporating CT from *A. mearnsii* as forage and extract, respectively. The greater in situ degradation and in vitro digestibility of DM, evidenced in the present study, is probably due to the absence (T1: 0%) or low content of tannins in the diet (T2: 3% CT), that is an amount less than 5% of tannins [52,53,54]. This would mean a reduction in the detrimental effects of CT on feed degradation and digestibility.

The low microbial biomass production evidenced in this study with T3 and T4 could be attributed to the amount of tannin in the experimental diets (64–95 g/kg DM), higher than the maximum levels that suppresses the negative effects of tannins on microbial rumen populations (<55 g/kg DM) [29]. This probably hindered the access of rumen microbes to plant cell wall protein or inhibited the growth and activity of fibrolytic bacteria capable of releasing the fibrolyzed protein, with a consequent reduction in the production of microbial protein [38,55]. The higher microbial biomass production evidenced in T2 is probably associated with its moderate CT content (<55 g/kg DM) and the *A. mearnsii* protein contribution which increased the efficiency of use from corn stover, thus increasing rumen microbial protein synthesis.

### 4.2. Rumen Protozoan Population

The decrease in ruminal protozoa (holotrich and entodiniomorph) could be a consequence of the CT content of diets which affected growth and activity of the methanogenic archaea and had a subsequent indirect effect on the population of protozoa. Moreover, due to the rupture of the symbiosis between these microorganisms, and consequent inhibition in the exchange of H_2_ between species (methanogen–protozoan), as a result of changes in cell membrane permeability, possibly due to CT acting on microbial adhesin [24,56,57,58]. Although holothrich protozoa have been reported to be more susceptible to tannins than entodiniomorph protozoa [59,60,61,62], a similar decrease was observed between the two species in the present study.

### 4.3. Gas, CH_4_ and CO_2_ Production

Gas, CH_4_ and CO_2_ production was influenced by the CT from *A. mearnsii*, showing a constant reduction in the production of CH_4_ as the amount of tannin rose. These effects are probably attributed to a reduction in methanogenic archaea and ruminal protozoa and formation of the tannin–fiber complex, which consequently reduces fiber degradation and digestibility [56,63]. Evidenced in the present study, was a limited degradation of the fiber, which probably decreased the availability of ruminal H_2_, in response to the reduction in the production of acetic acid from pyruvate [64]. H_2_ is necessary for methanogenic microorganisms for CH_4_ synthesis from CO_2_. This would explain the partial increase in CO_2_ at 24 h post-incubation with diet T2 due to reduced H_2_ availability at the ruminal level [65,66].

## 5. Conclusions

It is concluded that it is possible to replace corn stover with up to 15% of *A. mearnsii* (T2: 30.5 g CT/kg DM) since this decreased the population of ruminal protozoa (holotrich and entodiniomorph) as well as the production of CH_4_ in all hours evaluated, without generating adverse effects on ruminal digestion and microbial biomass production.

## Figures and Tables

**Table 1:** Experimental diets and chemical composition (g/kg DM).

Item	Treatments			
	T1	T2	T3	T4
Corn stover	1000	850	700	550
*A. mearnsii* leaves ^a^	0	150	300	450
Total	1000	1000	1000	1000
**Chemical composition**				
Dry matter	870.0	881.4	887.3	885.1
Organic matter	894.5	916.5	920.9	922.4
Crude protein	96.6	104.9	114.2	117.5
Neutral detergent fiber	658.9	619.9	567.8	542.5
Acid detergent fiber	360.3	341.6	325.5	312.8
Non-fiber carbohydrate	138.08	187.06	229.59	251.28
Fat	1.02	4.74	9.41	11.22
Ash	105.4	83.4	79.0	77.5
Condensed tannins	0	30.5	64.0	95.8

T1: 100% corn stover, T2: 85% corn stover + 15% *A. mearnsii* leaves, T3: 70% corn stover + 30% *A. mearnsii* leaves, T4: 65% corn stover + 45% *A. mearnsii* leaves, non-fiber carbohydrate = (1000 − (Crude protein + neutral detergent fiber + fat + ash)) estimated according to Getachew et al. (2004), ^a^: 188.7 g/kg DM of condensed tannins.

**Table 2:** Degradation kinetics, digestibility, microbial biomass production and rumen protozoa population in diets based on corn stover with increasing levels of *Acacia mearnsii* (g/kg DM, except where stated).

	T1	T2	T3	T4	SE	*p* Value
Degradation kinetics						
A	275.6 ^a^	291.9 ^a^	265.3 ^a^	273.2 ^a^	22.10	0.8710
B	530.0 ^a^	473.0 ^ab^	434.0 ^b^	412.0 ^b^	23.52	0.0137
A + B	806.0 ^a^	764.0 ^ab^	699.0 ^bc^	685.0 ^c^	18.31	0.0008
c	0.045 ^a^	0.039 ^a^	0.041 ^a^	0.034 ^a^	0.0067	0.7049
Effective degradation						
0.02 *k*	639.0 ^a^	598.0 ^b^	551.0 ^c^	513.0 ^d^	9.13	0.0001
0.05 *k*	524.0 ^a^	495.0 ^ab^	457.0 ^bc^	427.0 ^c^	9.84	0.0001
0.08 *k*	465.0 ^a^	444.0 ^ab^	410.0 ^bc^	388.0 ^c^	9.43	0.0001
IVDMD (%)	53.40 ^a^	49.76 ^ab^	46.08 ^b^	38.31 ^b^	1.84	0.0002
MBP (mg/0.5 g DM)	314.50 ^a^	321.10 ^a^	274.40 ^b^	216.30 ^c^	7.74	0.0001
Population of rumen protozoa						
Holotrich (Log_10_)						
12 h	3.8 ^a^	2.4 ^b^	1.5 ^c^	0.5 ^d^	0.12	0.0001
24 h	3.8 ^a^	1.9 ^b^	1.0 ^c^	0 ^d^	0.15	0.0001
Entodiniomorph (Log_10_)						
12 h	4.1 ^a^	3.5 ^b^	2.9 ^c^	1.6 ^d^	0.10	0.0001
24 h	4.0 ^a^	3.1 ^b^	2.2 ^c^	1.1 ^d^	0.14	0.0001

^a–d^ Means with different letters between columns differ significantly (*p* < 0.05), A: degradation of the soluble fraction, B: degradation of the insoluble but potentially degradable fraction, A + B: degradation potential, *c*: degradation rate in % per hour, *k*: rate of passage, IVDMD: in vitro dry matter digestibility, MBP: microbial biomass production (mg/0.5 g DM).

**Table 3:** Gas, CH_4_ and CO_2_ production characteristics of corn stover with increasing levels of *Acacia mearnsii*.

	T1	T2	T3	T4	SE	*p*-Value
Gas production parameters						
D	166.8 ^a^	168.4 ^a^	145.5 ^b^	117.8 ^c^	3.47	0.0001
*k*	0.038 ^c^	0.043 ^bc^	0.052 ^ab^	0.061 ^a^	0.0024	0.0001
Gas production (mL/0.5 g fermented DM)						
12 h	61.3 ^a^	68.9 ^a^	66.9 ^a^	66.0 ^a^	3.52	0.4958
24 h	96.8 ^a^	105.6 ^a^	97.9 ^a^	92.3 ^a^	3.97	0.1636
48 h	138.9 ^a^	148.7 ^a^	135.5 ^a^	120.3 ^b^	3.32	0.0002
CH_4_ production (mL/0.5 g fermented DM)						
12 h	3.5 ^a^	1.4 ^b^	1.0 ^b^	1.1 ^b^	0.61	0.0377
24 h	12.9 ^a^	10.3 ^b^	6.4 ^b^	6.7 ^b^	1.78	0.0415
48 h	22.6 ^a^	18.4 ^b^	12.7 ^c^	8.1 ^d^	1.01	<0.0001
CO_2_ production (mL/0.5 g fermented DM)						
12 h	29.8 ^a^	30.3 ^a^	25.2 ^a^	26.3 ^a^	2.14	0.2839
24 h	68.8 ^a^	71.5 ^a^	55.8 ^b^	45.6 ^c^	2.37	<0.0001
48 h	79.4 ^a^	85.1 ^a^	76.6 ^a^	82.2 ^a^	5.55	0.7255

^a–d^ Means with different letters between columns differ significantly (*p* < 0.05), D = potential cumulative gas production (mL/0.5 g fermented DM), *k* = rate of gas production (h).

## Data Availability

The data presented in this paper are available on request from the corresponding author.
